# Retraction: Evaluation of chronic nanosilver toxicity to adult zebrafish

**DOI:** 10.3389/fphys.2023.1282752

**Published:** 2023-10-13

**Authors:** Frontiers Editorial Office

The journal retracts the 2017 article cited above.

Following publication, concerns were raised regarding the integrity of the images in the published figures.

The authors failed to provide a satisfactory explanation during the investigation, which was conducted in accordance with Frontiers’ policies. As a result, the data and conclusions of the article have been deemed unreliable and the article has been retracted.

This retraction was approved by the Chief Editors of Frontiers in Physiology and the Editor-in-Chief of Frontiers. The authors have not agreed to the retraction.

